# Ceftriaxone use evaluation at the Medical Ward, Jigme Dorji Wangchuck National Referral hospital in Bhutan: A retrospective analytical study

**DOI:** 10.1016/j.ijregi.2025.100799

**Published:** 2025-11-04

**Authors:** Kezang Tshering, Mongal Singh Gurung, Sonam Wangda, Pelden Chejor

**Affiliations:** 1 Department of Pharmacy, Jigme Dorji Wangchuck National Referral Hospital, Thimphu, Bhutan; 2 Health Research and Epidemiology Unit, Policy and Planning Division, Ministry of Health, Thimphu, Bhutan; 3 Essential Medicine and Technology Division, Ministry of Health, Thimphu, Bhutan; 4 Center for Research in Aged Care, Edith Cowan University, Perth, Australia

**Keywords:** Ceftriaxone, Antibiotic, Drug use evaluation

## Abstract

**Objectives:**

Ceftriaxone is the most commonly prescribed cephalosporin antibiotic in hospital settings. The overall prescribing compliance with the national antibiotic guidelines remained unassessed at Jigme Dorji Wangchuk National Referral Hospital (JDWNRH), Bhutan.

**Methods:**

A retrospective analytical study was conducted by evaluating the medical records of patients who received ceftriaxone between January and December 2020 in the Medical Ward, JDWNRH. Data were analyzed using the Statistical Package for the Social Sciences, version 26.

**Results:**

A total of 201 (17.82%) patients, with a mean age of 54.83 years (SD ± 20.93), received ceftriaxone, and pneumonia (49.25%) was the most frequently documented indication. The overall appropriate use of ceftriaxone was observed among 37 patients (18.41%), while 47 cases (23.38%) were not assessable. Doxycycline (35.82%) and metronidazole (19.90%) were the most commonly co-prescribed antibiotics. Contraindications and serious drug-drug interactions were observed among 20 (9.95%) and 21 (10.45%) patients, respectively. The proportion of inappropriate use of ceftriaxone was significantly higher among patients who experienced a hospital stay of ≤7 days (*P* = 0.016).

**Conclusions:**

Routine monitoring and evaluation of ceftriaxone use as part of an antimicrobial stewardship program are recommended to prevent antibiotic resistance in the future.

## Highlights

• The overall appropriateness of ceftriaxone use was low in the Medical Ward, Jigme Dorji Wangchuk National Referral Hospital.
• Pneumonia was the most common indication for ceftriaxone use.
• Routine monitoring and evaluation are recommended to avoid misuse of ceftriaxone.
• Drug-interaction checkers should be adopted to prevent life-threatening adverse effects.

## Introduction

Antibiotics are among the most used and misused of all drugs [1,2]. Inappropriate use of antibiotics contributes to the emergence of resistant organisms [3]. Individuals infected with bacteria resistant to specific antibiotics are at increased risk of poor clinical outcomes and death [4]. Further, the emergence of resistant organisms such as methicillin-resistant *Staphylococcus aureus*, extended-spectrum beta-lactamase–producing *Escherichia coli* and *Klebsiella pneumoniae*, penicillin-resistant pneumococci, vancomycin-resistant *Enterococci*, and imipenem-resistant gram-negative bacilli has resulted in poor clinical outcomes, increased length of hospitalization, and higher treatment costs [5]. A World Health Organization (WHO) antimicrobial surveillance study across six regions found that more than half of *E. coli* and *K. pneumoniae* cases and over one-fourth of *Neisseria gonorrhoeae* cases were resistant to third-generation cephalosporins in the majority of surveyed regions [4].

Ceftriaxone is the most commonly prescribed third-generation cephalosporin owing to its wide spectrum of activity against bacterial pathogens and low toxicity [6,7]. The widespread use of ceftriaxone is attributed to its effectiveness against organisms causing pneumonia, urinary tract infection, bacterial meningitis, bacteremia/septicemia, skin and soft tissue infection, abdominal infection, bone infection, and acute bacterial otitis media. However, evidence has shown a high prevalence of inappropriate use of ceftriaxone across the globe [[8], [9], [10]].

Cephalosporins are frequently targeted for drug-use evaluation by antimicrobial stewardship programs due to the increasing trend in resistance associated with their irrational use. The defined daily dose of ceftriaxone increased from 82% in 2016 to 92% in 2017 at Jigme Dorji Wangchuck National Referral Hospital (JDWNRH) in Bhutan [11]. Despite the increased utilization of ceftriaxone at JDWNRH, the overall prescribing appropriateness of ceftriaxone remains questionable. Therefore, our study aimed to assess the overall prescribing compliance of ceftriaxone with the National Antibiotic Guidelines of Bhutan, 2018 (NAGoB-2018), and factors associated with inappropriate use of ceftriaxone at the Medical Ward of JDWNRH in Bhutan.

## Method

### Study design

A retrospective cross-sectional study was conducted to evaluate ceftriaxone utilization in the Medical Ward, JDWNRH, from January to December 2020.

### Setting

The study was conducted at JDWNRH, a tertiary-care hospital located in the western region of Bhutan. The hospital has a total bed capacity of 381 and provides free healthcare services to people across the country, ranging from basic healthcare services to complex cardiac care services. The Medical Ward under the Department of Medicine has a total of 38 beds, with an average monthly admission of 94 patients, as per the records maintained at the Medical Records Unit (MRU), JDWNRH.

### Materials

For overall appropriateness, ceftriaxone utilization was considered appropriate if all the documented categories, such as indication, dose, frequency, and duration, were correct according to NAGoB-2018. Ceftriaxone utilization was considered inappropriate if any one of the categories was found to be incorrect. For potential drug-drug interactions, the British National Formulary was used to identify both contraindications and potential drug interactions with ceftriaxone infusion.

### Data collection procedure

**Figure 1:**
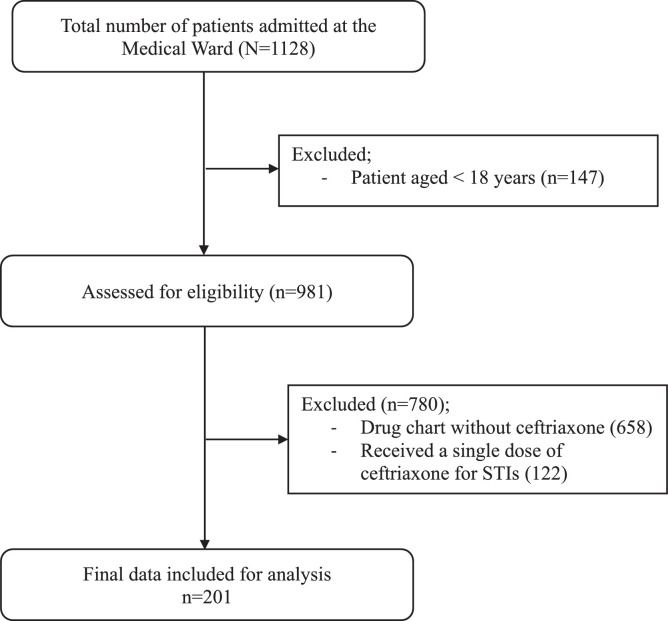
Data extraction and inclusion procedure to evaluate ceftriaxone utilization at the medical ward, Jigme Dorji Wangchuk National Referral Hospital between January to December 2020.

The medical records of all patients admitted to the Medical Ward were extracted from the database maintained at the MRU, JDWNRH. According to the database maintained at the MRU, a total of 1,128 patients were admitted to the Medical Ward during the study period. The overall processes involved in data extraction and inclusion are described in Figure 1.

The data collection questionnaire obtained demographic characteristics of patients such as age (<18, 18-65 and >65 years), sex (male/female), and clinical characteristics including the length of hospital stay (<7, 8-14, and >14 days), indications, dose, frequency, duration of treatment, antibiotics co-prescribed, contraindications (yes/no), and serious drug-drug interactions (yes/no).

### Inclusion and exclusion criteria

The medical records of patients who received ceftriaxone during the study period were included for evaluation and analysis. The medical records of patients who received a single dose of intramuscular (IM) ceftriaxone 250 mg for sexually transmitted diseases were excluded.

### Data analysis

Data were coded, entered, validated, and exported to SPSS version 26 for analysis. Descriptive analyses, such as frequency and percentage, were used to describe the overall appropriateness of ceftriaxone utilization. Chi-square and Fisher’s exact tests were used to assess differences in the proportion of inappropriate use of ceftriaxone among different categorical variables.

## Results

A total of 17.82% (201/1128) of patients received ceftriaxone in the Medical Ward, JDWNRH, during the study period. Patients’ age ranged from 13 to 96 years (mean 54.83 ± SD 20.9), with a similar proportion of male (48.76%) and female (51.24%) patients. More than half (55.22%) of the patients experienced hospital stays between 1 and 7 days. Ceftriaxone was commonly prescribed for pneumonia (49.25%). Nearly one-quarter (23.38%) of the ceftriaxone prescriptions were not assessable due to a lack of documented indications. The majority (86.57%) of the patients received ceftriaxone for up to 1 week, with a mean treatment duration of 5.16 days (SD ± 2.529). Doxycycline (35.82%) was the most commonly co-prescribed antibiotic, followed by metronidazole (19.90%). A contraindication (calcium gluconate) and serious drug-drug interactions (heparin and warfarin) were observed among 20 (9.95%) and 21 (10.45%) patients, respectively, as described in Table 1.

**Table 1:** Demographic and clinical characteristics of patients who received ceftriaxone at the Medical Ward, Jigme Dorji Wangchuk National Referral Hospital between January to December 2020.

Variables	Category	Frequency	Percentage
**Age (years)** (Mean age: 54.83 SD ± 20.93)	<18	4	1.99
	18-64	130	64.68
	≥65	67	33.33
**Sex**	Male	98	48.76
	Female	103	51.24
**Length of hospital stay (days)**	1-7	111	55.22
	8-14	57	28.36
	15-21	21	10.45
	≥22	12	5.97
**Indications**	Not assessable	47	23.38
	Pneumonia	99	49.25
	Septicemia	22	10.95
	Urinary tract infection	9	4.48
	Bacterial Meningitis	8	3.98
	Skin & soft-tissue infection	1	0.50
	Cardiovascular	1	0.50
	Gastrointestinal	6	2.99
	Others	8	3.98
**Duration of treatment received (days)**	0-7	174	86.57
	8-14	26	12.94
	>14	1	0.50
**Antibiotics co-prescribed**	None	65	32.34
	Doxycycline	72	35.82
	Metronidazole	40	19.90
	Doxycycline & metronidazole	7	3.48
	Azithromycin	5	2.49
	Ciprofloxacin	4	1.99
	Others	8	3.98
**Contraindication** (concomitant infusion with calcium gluconate)	Yes	20	9.95
	No	181	90.05
**Serious drug-drug interactions observed?**	No	180	89.55
	Warfarin	13	6.47
	Heparin	8	3.98

### Overall appropriateness of ceftriaxone utilization

**Figure 2:**
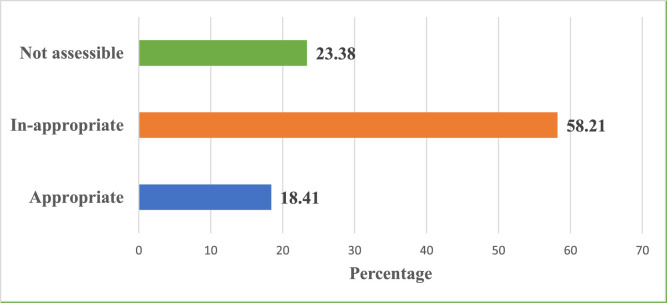
Overall appropriateness of ceftriaxone utilization at the medical ward, Jigme Dorji Wangchuk National Referral Hospital between January to December 2020.

**Figure 3:**
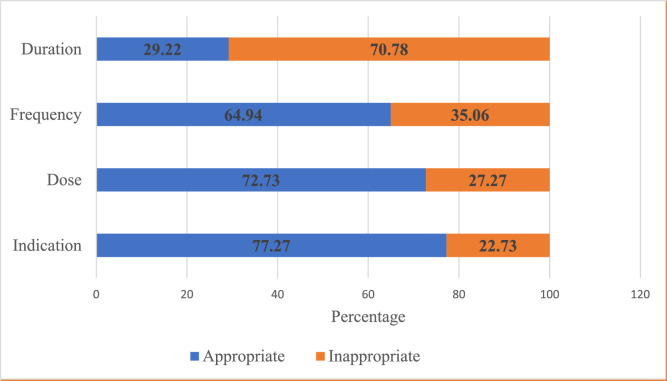
Overall percentage of appropriateness of ceftriaxone utilization by four indicators at the medical ward, Jigme Dorji Wangchuk National Referral Hospital between January to December 2020.

Using the four indicators of appropriate use, the overall appropriateness of ceftriaxone utilization was 18.41%. The majority (58.21%) of the patients received ceftriaxone inappropriately, as reflected in Figure 2. After assessing individual indicators, the highest level of appropriateness was observed for indication (77.27%), followed by dose (72.73%), frequency (64.94%), and duration of treatment (29.22%) (Figure 3).

### Factors associated with inappropriate use of ceftriaxone

Patients who experienced hospital stays of less than 7 days had significantly higher proportions of inappropriate ceftriaxone use *(P* = 0.016). No significant differences in the proportion of inappropriate ceftriaxone use were observed among different categories of age and sex in the study, as depicted in Table 2.

**Table 2:** Proportion of utilization of ceftriaxone at the medical ward between January to December 2020.

Variables	Appropriate	Inappropriate	*P-*value
	Frequency (%)	Frequency (%)	
**Age (years)**			
<18	0 (0.0)	3 (2.56)	0.242^a^
18-64	19 (51.35)	79 (67.52)	
≥65	18 (48.65)	35 (29.91)	
**Sex**			
Male	18 (48.65)	54 (46.15)	0.068
Female	19 (51.35)	63 (53.85)	
**Length of hospital stay (days)**			
1-7	13 (35.14)	70 (59.83)	0.016^a^
8-14	11 (29.73)	31 (26.50)	
15-21	10 (27.03)	8 (6.84)	
≥22	3 (8.11)	8 (6.84)	

a Fisher’s Exact Test.

## Discussion

The study was conducted to determine the overall appropriate use of ceftriaxone in the Medical Ward, JDWNRH, in compliance with NAGoB-2018. Of the 1128 admissions, 17.82% of patients received ceftriaxone during the study period. The overall prescribing incidence of ceftriaxone was higher than that reported in a study conducted at the medical ward of the national referral hospital in Eritrea (11.43%) [12]. In contrast, the incidence was much lower than in similar studies conducted at Gondar University Referral Hospital (59%) [8] and Tikur Anbessa Specialized Hospital (58%) in Ethiopia [13]. Such differences could be due to variations in the number of patients admitted annually because of infectious diseases and the overall bed capacity of the hospitals.

In our study, nearly half (49.3%) of ceftriaxone prescriptions were indicated for the treatment of pneumonia, consistent with prescribing trends reported in previous studies [10,[12], [13], [14]]. Pneumonia is the most common respiratory tract infection in patients of all ages. The excellent coverage of ceftriaxone against both gram-positive and gram-negative bacteria causing pneumonia, along with its low toxicity, are some of the major reasons leading to higher incidences of ceftriaxone use for pneumonia across hospitals.

Doxycycline (35.82%) and metronidazole (19.90%) were the two most common antibiotics co-prescribed with ceftriaxone. According to NAGoB 2018, ceftriaxone plus doxycycline is recommended for non-intensive care patients with poor response to ampicillin and doxycycline in combination. This indicates that more than one-third of the patients received ceftriaxone as an alternative therapy in combination with doxycycline. Metronidazole is another common antibiotic prescribed with ceftriaxone, considering its activity against anaerobic bacterial infections not covered by ceftriaxone. Moreover, metronidazole is preferred because it is cost-effective and possesses favorable pharmacokinetic and pharmacodynamic properties with minor adverse effects.

Concomitant infusion of ceftriaxone and calcium-containing solutions has caused infant mortality due to ceftriaxone-calcium precipitation [15]. Twenty (9.95%) patients in the study received ceftriaxone and calcium gluconate concomitantly; however, there was no evidence of mortality among non-infants. Furthermore, ceftriaxone-calcium precipitation occurs at a lower calcium concentration in neonatal plasma compared with adults. Despite no mortality being reported among patients older than 28 days, ceftriaxone and calcium-containing products may be administered sequentially, provided the infusion lines are properly flushed with compatible fluids between infusions. Similarly, a potential interaction was observed among 10.45% of the patients who were prescribed warfarin or heparin, which potentiates anticoagulant effects and increases the risk of bleeding. To prevent serious complications from drug-drug interactions, a clinical pharmacist should be involved in reviewing the prescriptions, or the hospital should adopt a drug-interaction checker system to detect and prevent any potential drug-drug interactions from reaching patients.

The overall appropriate use of ceftriaxone in the Medical Ward, JDWNRH (18.41%), was lower than in studies conducted at Kahsay Abera (61.75%) and Mearg (63.0%) hospitals in Ethiopia [16] and in Ayder Referral Hospital, Mekelle, Ethiopia (35.8%) [9]. Free access to essential medicines, including all antimicrobial agents in Bhutan as mandated by the Medicine Act of the Kingdom of Bhutan 2003, could be one major reason leading to irrational prescribing of antibiotics. This is supported by a recent meta-analysis whereby free healthcare services were linked to an 87.1% lower likelihood of compliance with guidelines [17]. Moreover, this may have been compounded by the lack of routine antimicrobial stewardship rounds in the Medical Ward, JDWNRH, by the infectious disease physician and the clinical pharmacist.

About one-quarter (23.38%) of the admissions were not assessable due to incomplete documentation. This indicates the importance of adopting a pre-authorization process in the hospital to necessitate documentation such as indication, dose, frequency, and duration when prescribing broad-spectrum antibiotics. According to the WHO Access, Watch, and Reserve classification system, ceftriaxone prescribing should be subject to constant monitoring and evaluation, for which complete documentation is essential to understand overall prescribing practices across hospitals. When analyzing individual indicators, the duration of treatment was found to be the least appropriate. Early antibiotic escalation owing to poor clinical response could be the major cause of low compliance with NAGoB 2018 in terms of the duration of ceftriaxone therapy.

A significantly higher proportion of patients who stayed in the hospital between 1 and 7 days displayed inappropriate use of ceftriaxone compared with patients who stayed more than 7 days. A similar observation was reported in a recent study, where inappropriate use was 83.3% and 78% less likely among patients who experienced hospital stays between 8 and 14 days and greater than 14 days, respectively, compared with hospital stays of 7 days and below [17]. No significant differences in inappropriate use of ceftriaxone were observed among different sex and age categories in the study.

## Conclusion

Our study concluded that the overall appropriateness of ceftriaxone use was low in the Medical Ward, JDWNRH. This indicates the risk of the emergence of antibiotic resistance in the near future, necessitating broader-spectrum antibiotics to manage infectious diseases caused by resistant organisms such as extended-spectrum beta-lactamase-producing organisms. Therefore, the use of ceftriaxone should be constantly monitored by antimicrobial stewardship programs, consisting of infectious disease physicians and clinical pharmacists, using a pre-authorization process, and approved only for confirmed bacterial infections guided by the local susceptibility pattern. Moreover, preventing the emergence of multidrug-resistant organisms (MDROs) through the rational use of antibiotics such as ceftriaxone should be considered a hospital priority to minimize the risk of morbidity and mortality associated with MDROs in the future.

### Limitations

A retrospective design of the study, in which 23.38% of the medical records were not assessable due to incomplete documentation, was one major limitation. Including other major departments, such as surgical and emergency, would have provided better representativeness of the overall ceftriaxone use at JDWNRH. Our study was not able to present information on culture and susceptibility reports due to the lack of a standard report-sharing system between the Medical Ward and the Microbiology Unit at JDWNRH.

## Declaration of competing interest

The authors have no competing interests to declare.

## Appendix Supplementary materials

Image, application 1
